# Treatment of plaque psoriasis with an ointment formulation of the Janus kinase inhibitor, tofacitinib: a Phase 2b randomized clinical trial

**DOI:** 10.1186/s12895-016-0051-4

**Published:** 2016-10-03

**Authors:** Kim A. Papp, Robert Bissonnette, Melinda Gooderham, Steven R. Feldman, Lars Iversen, Jennifer Soung, Zoe Draelos, Carla Mamolo, Vivek Purohit, Cunshan Wang, William C. Ports

**Affiliations:** 1 K Papp Clinical Research and Probity Medical Research Inc, Waterloo, ON Canada; 2 Innovaderm Research, Montreal, QC Canada; 3 SKiN Centre for Dermatology and Probity Medical Research Inc, Peterborough, and Queens University, Kingston, ON Canada; 4 Wake Forest Baptist Health, Winston-Salem, NC USA; 5 Aarhus University Hospital, Aarhus, Denmark; 6 Southern California Dermatology, Santa Ana, CA USA; 7 Dermatology Consulting Services, High Point, NC USA; 8 Pfizer Worldwide Biopharmaceuticals, Global Innovative Pharma Business, Groton, CT USA

**Keywords:** Psoriasis, Topical, Tofacitinib, CP-690,550, Physician’s Global Assessment, Psoriasis Area and Severity Index, PASI, Dermatology Life Quality Index, Pruritus, Itch

## Abstract

**Background:**

Most psoriasis patients have mild to moderate disease, commonly treated topically. Current topical agents have limited efficacy and undesirable side effects associated with long-term use. Tofacitinib is a small molecule Janus kinase inhibitor investigated for the topical treatment of psoriasis.

**Methods:**

This was a 12-week, randomized, double-blind, parallel-group, vehicle-controlled Phase 2b study of tofacitinib ointment (2 % and 1 %) applied once (QD) or twice (BID) daily in adults with mild to moderate plaque psoriasis. Primary endpoint: proportion of patients with Calculated Physician’s Global Assessment (PGA-C) clear or almost clear and ≥2 grade improvement from baseline at Weeks 8 and 12. Secondary endpoints: proportion of patients with PGA-C clear or almost clear; proportion achieving Psoriasis Area and Severity Index 75 (PASI75) response; percent change from baseline in PASI and body surface area; change from baseline in Itch Severity Item (ISI). Adverse events (AEs) were monitored and clinical laboratory parameters measured.

**Results:**

Overall, 435 patients were randomized and 430 patients received treatment. The proportion of patients with PGA-C clear or almost clear and ≥2 grade improvement from baseline at Week 8 was 18.6 % for 2 % tofacitinib QD (80 % confidence interval [CI] for difference from vehicle: 3.8, 18.2 %) and 22.5 % for 2 % tofacitinib BID (80 % CI: 3.1, 18.5 %); this was significantly higher vs vehicle for both dosage regimens. No significant difference vs vehicle was seen at Week 12. Significantly more patients achieved PGA-C clear or almost clear with 2 % tofacitinib QD and BID and 1 % tofacitinib QD (not BID) at Week 8, and with 2 % tofacitinib BID at Week 12. Pruritus was significantly reduced vs vehicle with 2 % and 1 % tofacitinib BID (starting Day 2), and 2 % tofacitinib QD (starting Day 3). Overall, 44.2 % of patients experienced AEs, 8.1 % experienced application site AEs, and 2.3 % experienced serious AEs. The highest incidence of AEs (including application site AEs) was in the vehicle QD group.

**Conclusions:**

In adults with mild to moderate plaque psoriasis, 2 % tofacitinib ointment QD and BID showed greater efficacy than vehicle at Week 8, but not Week 12, with an acceptable safety and local tolerability profile.

**Trial registration:**

NCT01831466 registered March 28, 2013.

**Electronic supplementary material:**

The online version of this article (doi:10.1186/s12895-016-0051-4) contains supplementary material, which is available to authorized users.

## Background

The World Health Organization has described psoriasis as a ‘chronic, non-communicable, painful, disfiguring and disabling disease for which there is no cure’ [1]. The majority of people with plaque psoriasis (75–90 %) are considered to have relatively limited mild to moderate disease [2, 3]. Many treatments are available for mild to moderate psoriasis, including topical treatment with corticosteroids, often in combination with vitamin D analogues [4–6]. The use of mid to high potency corticosteroids can be limited by local and systemic adverse effects, particularly on the face and intertriginous areas [5, 7, 8]. Irritation or burning can also occur with vitamin D analogues [9–11]. Topical therapy is also used in combination with phototherapy or systemic therapy in patients with moderate to severe psoriasis [12].

A substantial proportion of patients with psoriasis are dissatisfied with their current treatment [13]. The limited efficacy of non-steroidal topical monotherapy or low potency corticosteroids and the safety issues associated with long-term use of mid to high potency topical corticosteroids suggest an unmet need exists for additional topical therapeutic options.

Tofacitinib (CP-690,550) is a small molecule Janus kinase (JAK) inhibitor; inhibition of JAK1 and JAK3 by tofacitinib blocks signaling of multiple cytokines implicated in immune response and inflammation. The oral formulation of tofacitinib is effective in patients with moderate to severe plaque psoriasis [14–16]. An ointment formulation of tofacitinib investigated for the topical treatment of psoriasis in a Phase 2a study showed the ointment (2 % twice daily [BID]) was effective with acceptable tolerability for mild to moderate psoriasis [17].

The primary objective of this Phase 2b study was to further characterize the efficacy and safety of tofacitinib ointment (2 % and 1 %) applied once daily (QD) or BID over 12 weeks in adult patients with mild or moderate chronic plaque psoriasis, compared with the corresponding vehicle.

## Methods

### Study design and treatment

This randomized, double-blind, parallel-group, vehicle-controlled study (NCT01831466), conducted at 52 centers in the United States, Canada, Denmark, and Poland, was initiated in May 2013 and completed in September 2014. Patients were randomized 1:1:1 to receive 1 % (10 mg/g) tofacitinib ointment, 2 % (20 mg/g) tofacitinib ointment or corresponding vehicle. Randomization was stratified by baseline severity of psoriasis as defined by the Calculated Physician’s Global Assessment (PGA-C). Investigators, study staff and sponsor remained blinded to treatment and randomization information until after the conclusion of the study. Investigator sites were assigned to either QD or BID regimen, but not both; neither investigators nor patients were blinded to regimen.

Tofacitinib ointment was provided in 60 g tubes at a strength of 2 % (maximum feasible concentration) and 1 %; the matching vehicle contained the same inactive ingredients as tofacitinib ointment. Treatments were administered topically at a target application coverage of 3 mg/cm^2^ to a treatment area corresponding to 2 to 20 % of the patient’s body surface area (BSA). Patients were instructed to treat all treatment-eligible psoriatic areas identified at baseline for 12 weeks, regardless of clearing or improvement in psoriasis. On study visit days, showering or bathing, but not moisturizing, was permitted prior to attending, and study drug was applied in the clinic after study assessments were completed. After the final study treatment, the treatment areas were left untreated during the 4-week follow-up period.

Use of shampoo containing tar, salicylic acid or low or least potent corticosteroid products (eg hydrocortisone and hydrocortisone acetate ≤1 %) was permitted on hair-bearing scalp only throughout the study. The proprietary ointment formulation contained standard excipients for a topical formulation.

### Patients

#### Key inclusion criteria

Subjects were aged ≥18 years with chronic plaque psoriasis for ≥6 months, were required to have a PGA-C score of mild (2) or moderate (3), and have plaque psoriasis covering 2–20 % of their BSA on the trunk and/or limbs, with ≥1 % BSA involvement on the trunk and/or limbs (excluding palms, soles, elbows, knees and below the knees).

#### Key exclusion criteria

Exclusion criteria included non-plaque forms of psoriasis; drug-induced psoriasis; evidence of skin conditions that would interfere with the evaluation of psoriasis; history of infection requiring hospitalization or treatment with oral or topical antimicrobial therapy within 2 weeks prior to baseline; hepatitis B/C or HIV infection; history of lymphoproliferative disorder or malignancy, except adequately treated or excised basal/squamous cell carcinoma, or cervical carcinoma in situ; evidence of tuberculosis infection; treatment with ustekinumab within the previous 4 months or other biologic agents (excluding etanercept) within the previous 2 months; phototherapy or treatment with etanercept or conventional systemic treatments that could affect psoriasis, such as oral or injectable corticosteroids, retinoids, methotrexate, and cyclosporine, within 4 weeks prior to the first study dose.

Topical treatments that could affect psoriasis (eg corticosteroids, tars, keratolytics, anthralin, vitamin D analogues, and retinoids) were discontinued for ≥2 weeks prior to the first study dose.

### Assessments

Clinical signs of plaque psoriasis (erythema, induration and scaling) were scored separately according to a 5-point severity scale: clear (0), almost clear (1), mild (2), moderate (3), and severe (4). These PGA subscores were then summed, averaged, and rounded to the nearest whole number to determine the PGA-C score and category [18]. Evaluation of the PGA-C excluded the scalp (even if the hairless scalp was being treated with study drug), palms, soles, and nails.

The primary endpoint was the proportion of subjects achieving a PGA-C response of clear (0) or almost clear (1) with ≥2 grade improvement from baseline at Week 8 and Week 12, independently. Secondary endpoints included Week 8 and Week 12 assessments of the proportion of patients achieving a PGA-C response of clear (0) or almost clear (1); the proportion of patients achieving a ≥75 % improvement from baseline in Psoriasis Area and Severity Index (PASI75); the percent change from baseline in PASI; and the percent change from baseline in affected BSA.

Evaluation of patient-reported outcomes included change from baseline in itch severity and in the Dermatology Life Quality Index (DLQI). The severity of itch was assessed via the Itch Severity Item (ISI), a single item instrument in which the patient records itching over the previous 24 h on a numerical rating scale of 0 (no itching) to 10 (worst possible itching) [19]. ISI was recorded in the clinic during Visit 1 (baseline/Day 1) and at Visits 3–7 (Weeks 2, 4, 8, 12, and 16), as well as once per day between Visit 1 and the day before Visit 3 by the patient in a diary prior to application of study treatment. Patients in the BID treatment group recorded the ISI before applying either the morning or evening treatment, but at the same time throughout this period.

Safety endpoints included the incidence of treatment-emergent adverse events (AEs), serious AEs (SAEs), and application site AEs, plus the proportion of patients who discontinued due to application site AEs. Physical examination, monitoring of vital signs, and clinical laboratory assessments (including hematology, fasting serum chemistry, fasting lipid panels, and urinalysis) were performed.

Pharmacokinetic (PK) endpoints included tofacitinib PK concentrations for pre-dose and post-dose samples. Pre-dose blood samples were collected at baseline and at Weeks 2, 4, 8, and 12 (0 h). At selected sites, three PK samples were also collected at Week 4 post-dose between 30 min and 1 h, between 2 and 3 h, and between 4 and 10 h.

### Statistical analysis

This was an estimation study. A sample size of 70 subjects per treatment group was selected, such that the 80 % confidence interval (CI) width of the difference between tofacitinib and vehicle was approximately 19 %, assuming a 21 % vehicle response and a 36 % response in tofacitinib. Additionally, this sample size would yield approximately 76 % power to establish the superiority of each strength and regimen of tofacitinib to its respective vehicle for the primary endpoint at the 0.10 (one-sided) significance level. No adjustment for multiple comparisons was made.

Patients with mild or moderate psoriasis at baseline (as defined by PGA-C) who were randomized and received at least one dose of study medication (tofacitinib or vehicle) were included in the analyses. Data at Week 8 and Week 12 were evaluated separately.

For the primary endpoint, standard error (SE) and two-sided 80 % CI were calculated using the normal approximation to the binomial proportions. A stratified analysis was conducted by summarizing the difference in proportions adjusted for the baseline PGA-C disease severity using the Cochran-Mantel-Haenszel approach [20, 21]. Patients with missing values were considered non-responders.

PASI75 and PGA-C response of clear or almost clear were analyzed using a marginal logistic regression model fit by pseudo-likelihood (generalized linear mixed model for repeated measures). Response proportions were estimated from the model and odds ratios for treatment contrasts along with 80 % CI were determined. Continuous variables (eg percent change from baseline in PASI and BSA, and change from baseline in ISI) were analyzed using a linear mixed model for repeated measures. Least squares mean (LSM), difference in LSM, SE, and two-sided 80 % CI were calculated. All analyses used observed data without imputation. Separate models were fit for the QD and BID data.

For comparisons in response proportions between the active treatment and corresponding vehicle, statistical significance was declared if the lower limit of the two-sided 80 % CI for the response difference was >0 for the primary efficacy endpoint, and if the lower limit of the two-sided 80 % CI for the odds ratio was >1 for the secondary PGA-C and PASI75 endpoints. For comparisons in LSMs between active treatment and corresponding vehicle, statistical significance was declared if the upper limit of the two-sided 80 % CI was <0 for percent change from baseline in PASI and BSA and change from baseline in ISI. No adjustment for multiple comparisons was made.

All statistical analyses were performed using SAS Software [22].

## Results

### Patients

**Fig. 1:**
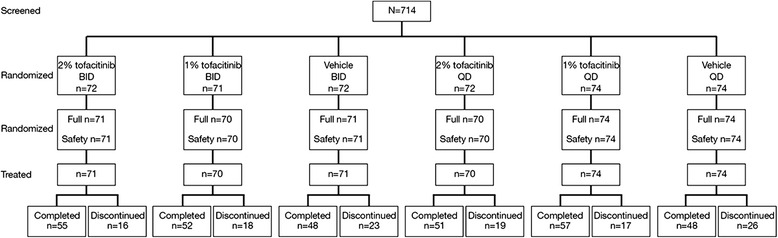
Patient disposition. Note that the 714 subjects includes subjects with mild, moderate, and severe psoriasis; subjects randomized are those that met the mild to moderate psoriasis eligibility criteria. Following database release, it was discovered that five subjects were randomized, but did not have any record of study drug dosing and were assessed in the clinical database as non-treated subjects; as a consequence, these subjects were excluded from all analyses. *BID* twice daily, *QD* once daily

Overall, 435 patients were randomized (Fig. 1). In the QD treatment groups, 218 patients received either 2 % tofacitinib, 1 % tofacitinib, or vehicle (*n* = 70, 74, 74, respectively). In the BID treatment groups, 212 patients received either 2 % tofacitinib, 1 % tofacitinib, or vehicle (*n* = 71, 70, 71, respectively). Baseline demographics were generally similar across the treatment groups, with the exception of geographical distribution between the dosing regimens (Table 1).

**Table 1:** Baseline patient demographics and disease characteristics

	2 % tofacitinib BID	1 % tofacitinib BID	Vehicle BID	2 % tofacitinib QD	1 % tofacitinib QD	Vehicle QD
	*N* = 71	*N* = 70	*N* = 71	*N* = 70	*N* = 74	*N* = 74
Age (years)						
Mean (SD)	47.6 (15.6)	50.4 (14.5)	48.8 (15.0)	50.7 (13.2)	47.8 (14.0)	48.9 (13.9)
Range	18.0–74.0	18.0–77.0	21.0–84.0	21.0–77.0	20.0–85.0	20.0–74.0
Male (%)	60.6	67.1	57.7	52.9	67.6	56.8
BMI (kg/m^2^)						
Mean (SD)	31.8 (7.9)	29.6 (5.6)	30.2 (8.3)	28.9 (7.8)	31.0 (7.2)	31.1 (6.5)
Range	17.2–58.4	19.2–51.4	17.0–79.8	16.4–68.6	17.6–50.4	20.6–47.4
Race (%)						
White	93.0	85.7	94.4	90.0	91.9	97.3
Black	1.4	4.3	1.4	1.4	2.7	2.7
Asian	4.2	7.1	2.8	5.7	2.7	0.0
Other	1.4	2.9	1.4	2.9	2.7	0.0
Geographical region (%)						
Canada	15.5	30.0	26.8	27.1	23.0	33.8
Denmark	1.4	1.4	0.0	1.4	1.4	1.4
Poland	14.1	12.9	15.5	32.9	39.2	28.4
United States	69.0	55.7	57.7	38.6	36.5	36.5
PGA-C (%)						
Mild	28.2	30.0	29.6	32.9	27.0	27.0
Moderate	71.8	70.0	70.4	67.1	73.0	73.0
PASI score						
Mean (SD)	9.5 (5.1)	8.5 (3.3)	8.5 (3.6)	9.9 (4.1)	10.1 (4.4)	9.6 (3.8)
Range	2.4–29.0	3.0–18.0	2.4–18.0	2.0–19.8	2.8–19.8	3.2–17.1
BSA (%)						
Mean (SD)	7.6 (4.6)	6.4 (3.8)	6.5 (4.1)	7.8 (4.3)	8.4 (4.9)	8.0 (4.5)
Range	2.0–19.0	1.5–17.0	2.0–20.0	2.0–19.0	2.4–20.0	2.0–19.0
ISI score^a^						
Mean (SD)	5.8 (2.6)	5.3 (2.4)	5.4 (2.6)	6.0 (2.7)	5.7 (2.9)	5.4 (3.0)
Range	0.0–10.0	1.0–10.0	0.0–10.0	0.0–10.0	0.0–10.0	0.0–10.0
DLQI						
Mean (SD)	10.6 (5.9)	8.6 (5.5)	9.3 (6.0)	12.2 (7.4)	10.9 (7.0)	10.2 (6.5)
Range	0.0–25.0	1.0–25.0	1.0–24.0	1.0–29.0	1.0–29.0	0.0–26.0

^a^Two patients were missing baseline ISI scores (1 in 2 % tofacitinib QD; 1 in 1 % tofacitinib BID)

*BID* twice daily, *BMI* body mass index, *BSA* body surface area, *DLQI* Dermatology Life Quality Index, *ISI* Itch Severity Item, *PASI* Psoriasis Area and Severity Index, *PGA*-*C* Calculated Physician’s Global Assessment, *QD* once daily, *SD* standard deviation

### Efficacy

Only those treatment groups and time points that were statistically significant are described within the text.

#### Primary endpoints

**Fig. 2:**
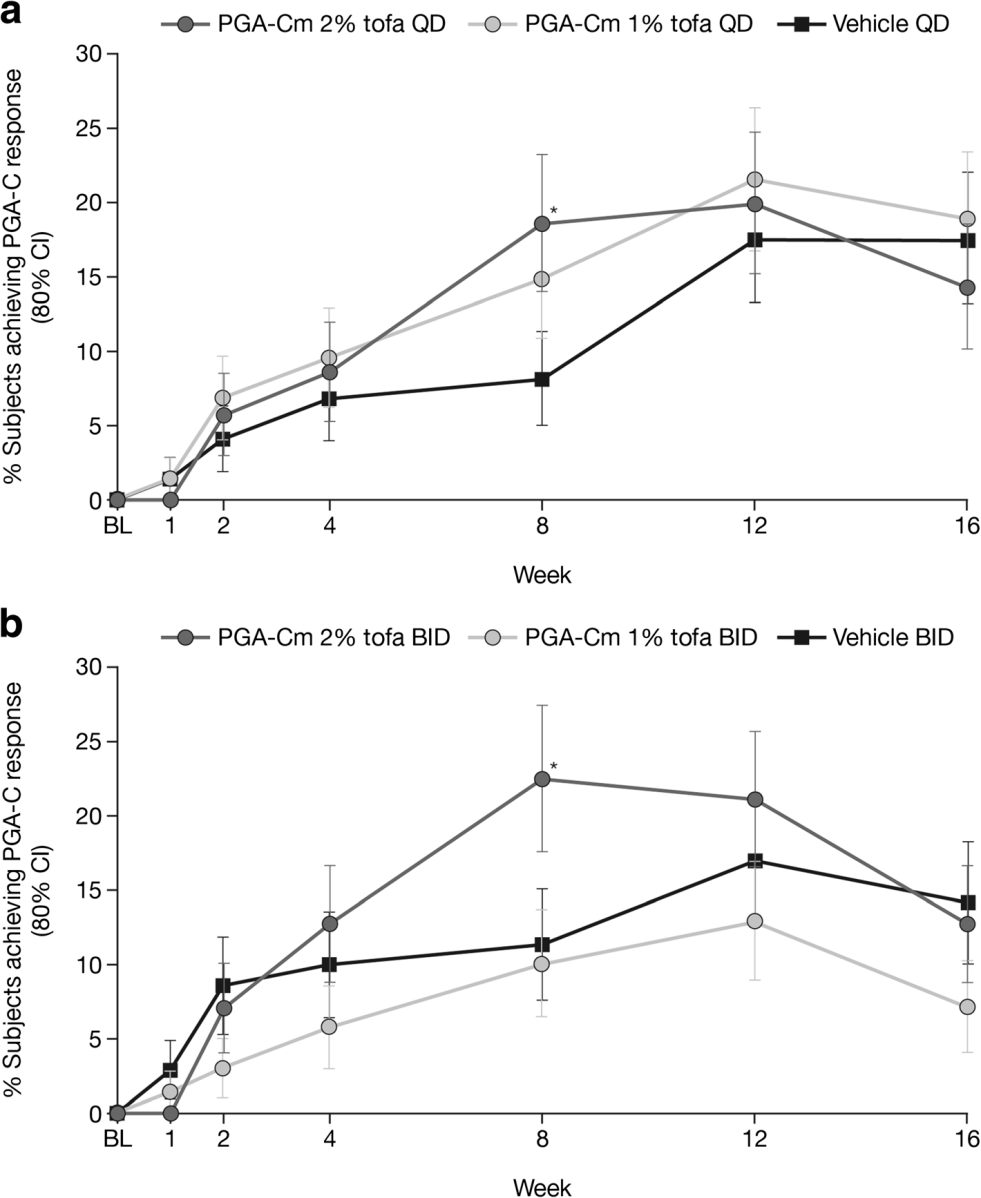
PGA-C response of clear (0)/almost clear (1) and ≥2 grade improvement at Week 16. *Lower limit 80 % CI of difference tofacitinib versus vehicle >0. Proportion (SE) of patients achieving a PGA-C response of clear (0) or almost clear (1) and ≥2 grade improvement from baseline through to Week 16 for patients applying 2 % tofacitinib, 1 % tofacitinib, or vehicle, once daily (**a**) or twice daily (**b**). Patients who were discontinued or with missing values were considered non-responders. *BID* twice daily, *BL* baseline, *CI* confidence interval, *PGA-C* Calculated Physician’s Global Assessment, *PGA-Cm* Calculated Physician’s Global Assessment of patients with mild to moderate plaque psoriasis at baseline, *QD* once daily, *SE* standard error, *tofa* tofacitinib

At Week 8 only, significantly more patients receiving 2 % tofacitinib QD and 2 % tofacitinib BID achieved a PGA-C response of clear or almost clear and ≥2 grade improvement from baseline compared with the corresponding vehicle. Response rate was 18.6 % and 8.1 % for 2 % tofacitinib QD and vehicle QD, respectively, and 22.5 % and 11.3 % for 2 % tofacitinib BID and vehicle BID, respectively. The difference (80 % CI) between response to active treatment and vehicle was 10.8 % (3.1, 18.5) and 11.0 % (3.8, 18.2) for 2 % tofacitinib BID and QD administration, respectively (Fig. 2a–b). At Week 12, no statistically significant differences versus vehicle were seen for 2 % or 1 % tofacitinib by either dosing regimen (Fig. 2a–b).

#### Secondary endpoints

The proportion of patients achieving a PGA-C response of clear or almost clear was significantly greater for the 2 % tofacitinib QD (35.9 %), 2 % tofacitinib BID (41.8 %) and 1 % tofacitinib QD (23.4 %) treatment groups compared with vehicle (QD 13.8 %, BID 25.2 %) at Week 8, and for the 2 % tofacitinib BID (39.7 %) treatment group compared with vehicle (27.3 %) at Week 12 (Table 2).

**Table 2:** Secondary efficacy endpoints

Endpoint	Week	2 % tofacitinib BID	1 % tofacitinib BID	Vehicle BID	2 % tofacitinib QD	1 % tofacitinib QD	Vehicle QD
PGA-C, clear (0) or almost clear (1) Responders, % (n/N) Odds ratios (80 % CI)	8	41.8 (26/61) 2.13^b^ (1.29, 3.54)	20.9 (14/64) 0.79 (0.45, 1.36)	25.2 (16/55)	35.9 (23/60) 3.52^b^ (1.97, 6.28)	23.4 (16/67) 1.92^b^ (1.06, 3.48)	13.8 (9/58)
	12	39.7 (24/58) 1.75^b^ (1.06, 2.90)	28.4 (18/57) 1.06 (0.62, 1.78)	27.3 (17/55)	36.1 (19/53) 1.36 (0.81, 2.28)	32.9 (22/62) 1.18 (0.71, 1.97)	29.3 (17/52)
PASI75 Responders, % (n/N) Odds ratios (80 % CI)	8	15.2 (10/61) 2.06 (0.96, 4.45)	9.1 (6/64) 1.15 (0.50, 2.66)	8.0 (5/55)	17.9 (11/60) 2.40^b^ (1.14, 5.05)	7.2 (5/67) 0.85 (0.36, 2.01)	8.3 (5/58)
	12	20.3 (12/58) 1.42 (0.76, 2.64)	14.4 (9/57) 0.94 (0.48, 1.83)	15.2 (9/55)	23.0 (11/53) 3.11^b^ (1.47, 6.55)	12.1 (8/62) 1.44 (0.65, 3.19)	8.8 (5/52)
PASI, % change from baseline LSM, % (N) Difference^a^ (80 % CI)	8	−31.8 (61) −8.3 (−17.3, 0.6)	−26.7 (64) −3.2 (−12.1, 5.7)	−23.5 (55)	−28.3 (60) −9.2^b^ (−17.1, −1.4)	−25.5 (67) −6.4 (−14.1, 1.3)	−19.1 (58)
	12	−33.9 (58) −6.5 (−17.1, 4.1)	−32.6 (57) −5.2 (−15.7, 5.3)	−27.4 (55)	−33.4 (53) −12.3^b^ (−21.8, −2.8)	−27.0 (62) −5.9 (−15.2, 3.3)	−21.1 (52)
BSA, % change from baseline LSM, % (N) Difference^a^ (80 % CI)	8	−22.1 (61) −4.2 (−13.1, 4.7)	−20.9 (64) −3.0 (−11.8, 5.8)	−17.9 (55)	−12.5 (60) −8.0 (−16.7, 0.7)	−10.1 (67) −5.7 (−14.2, 2.8)	−4.5 (58)
	12	−31.2 (58) −4.5 (−13.8, 4.9)	−26.0 (57) 0.8 (−8.5, 10.0)	−26.7 (55)	−22.8 (53) −20.0^b^ (−31.4, −8.7)	−12.3 (62) −9.5 (−20.6, 1.6)	−2.8 (52)

^a^Difference active – vehicle; ^b^meets specification for statistical significance

PASI excluded the scalp, palms, and soles from the assessment/scoring, even if these areas were being treated with study drug. BSA excluded the head, neck, palms, and soles, even if these areas were being treated with study drug

PGA-C and PASI75 responses were analyzed using a Generalized Mixed Model for Repeated Measures without imputation for missing values; percent changes from baseline in PASI and BSA were analyzed using a Mixed Model for Repeated Measures without imputation for missing values; QD and BID data were analyzed separately

*BID* twice daily, *BSA* body surface area, *CI* confidence interval, *LSM* least squares mean, *PASI* Psoriasis Area and Severity Index, *PGA*-*C* Calculated Physician’s Global Assessment, *QD* once daily

At Week 8 and Week 12, significantly more patients receiving 2 % tofacitinib QD (17.9 % and 23.0 %, respectively) achieved a PASI75 response vs vehicle (8.3 % and 8.8 %, respectively) (Table 2). The percent change from baseline in PASI was also significantly greater for the 2 % tofacitinib QD treatment group compared with vehicle at Week 8 and Week 12 (Table 2); the differences (80 % CI) vs corresponding vehicle were −9.2 % (−17.1, −1.4) and −12.3 % (−21.8, −2.8) at Weeks 8 and 12, respectively. The percent change from baseline in BSA was also significantly greater for the 2 % tofacitinib QD treatment group compared with vehicle at Week 12 (Table 2); the difference (80 % CI) vs corresponding vehicle was −20.0 % (−31.4, −8.7).

#### Patient-reported outcomes

**Fig. 3:**
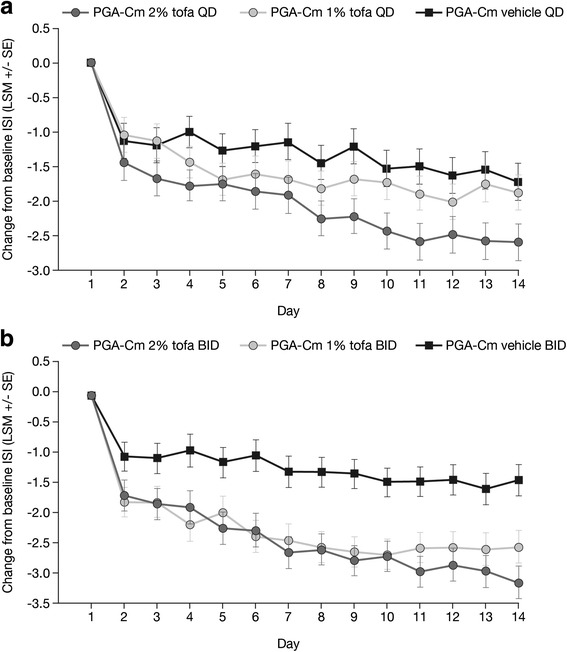
Change from baseline in Itch Severity Item score through Week 2. Least squares mean (SE) change from baseline in Itch Severity Item score through Week 2 for patients applying 2 % tofacitinib, 1 % tofacitinib, or vehicle, once daily (**a**) or twice daily (**b**). Changes from baseline in ISI were analyzed using a Mixed Model for Repeated Measures without imputation for missing values; QD and BID data were analyzed separately. *BID* twice daily, *ISI* Itch Severity Item, *LSM* least squares mean, *PGA-Cm* Calculated Physician’s Global Assessment of patients with mild to moderate plaque psoriasis at baseline, *QD* once daily, *SE* standard error, *tofa* tofacitinib

2 % and 1 % tofacitinib BID significantly reduced pruritus compared with vehicle BID as early as Day 2 (the day following the initial dose); these improvements were sustained through Day 14 (Fig. 3a). Numerically greater improvements in ISI were also seen in the 2 % and 1 % tofacitinib QD treatment groups compared with vehicle QD; these improvements were statistically significant for 2 % tofacitinib QD on Days 3–14 (Fig. 3b). Significant improvements in pruritus were maintained for 2 % BID, 1 % BID, and 2 % QD from Week 2 through Week 12 (except Week 8 and 12 for 2 % QD).

At Week 8, 2 % tofacitinib BID and QD significantly improved DLQI more than their respective vehicles (Additional file 1: Figure S1). At Week 12, 2 % tofacitinib QD and 1 % tofacitinib QD significantly improved DLQI more than the vehicle (Additional file 1: Figure S1).

### Safety

#### All adverse events

Overall, 44.2 % of patients experienced treatment-emergent AEs, most of which were mild or moderate in severity (Table 3). The highest incidence of treatment-emergent AEs was in the vehicle QD group, with 54.1 % of patients in this group reporting one or more treatment-emergent AE. The most frequently reported AEs by Medical Dictionary for Regulatory Activities (MedDRA; version 17.1) preferred term were nasopharyngitis (6.7 %), upper respiratory tract infection (4.9 %), and psoriasis (4.9 %).

**Table 3:** Summary of adverse events, patients discontinued due to adverse events and deaths

	2 % tofacitinib BID	1 % tofacitinib BID	Vehicle BID	2 % tofacitinib QD	1 % tofacitinib QD	Vehicle QD
	*N* = 71	*N* = 70	*N* = 71	*N* = 70	*N* = 74	*N* = 74
Number of AEs	47	51	54	66	65	62
Patients with treatment-emergent AEs, n (%)	30 (42.3)	30 (42.9)	28 (39.4)	34 (48.6)	28 (37.8)	40 (54.1)
Patients with application site AEs, n (%)	4 (5.6)	0 (0.0)	4 (5.6)	8 (11.4)	7 (9.5)	12 (16.2)
Patients with SAEs, n (%)	0 (0.0)	5 (7.1)	2 (2.8)	0 (0.0)	2 (2.7)	1 (1.4)
Patients discontinued due to AEs, n (%)	0 (0.0)	1 (1.4)^a^	4 (5.6)	6 (8.6)	3 (4.1)	7 (9.5)
Deaths, n (%)	0 (0.0)	1 (1.4)^a^	0 (0.0)	0 (0.0)	0 (0.0)	0 (0.0)

^a^Patient had an AE of myocardial infarction and subsequently died; the patient is counted as a discontinuation due to AE and as a death

Categories of adverse events experienced include treatment-emergent, application site and serious adverse events

*AE* adverse event, *BID* twice daily, *QD* once daily, *SAE* serious adverse event

A total of 11 SAEs were experienced by 10 (2.3 %) patients. No SAEs were reported in the 2 % tofacitinib QD or BID treatment groups; SAEs were reported in five patients in the 1 % tofacitinib BID group, in two patients in each of the 1 % tofacitinib QD and vehicle BID groups, and in one patient in the vehicle QD group (Additional file 1: Table S1). No SAEs were assessed by the investigator as treatment-related, with the exception of one SAE of psoriatic arthropathy in the vehicle BID treatment group.

Overall, 21 (4.9 %) patients discontinued from the study due to AEs, most commonly psoriasis, which was reported by six (1.4 %) patients. Seven patients discontinued due to AEs in the vehicle QD group, six with 2 % tofacitinib QD, four with vehicle BID, three with 1 % tofacitinib QD, and one with 1 % tofacitinib BID (patient was discontinued from the study due to a fatal myocardial infarction as described below).

One death (due to myocardial infarction) occurred in a 53-year-old white male receiving 1 % tofacitinib BID. His final application of tofacitinib ointment was on Study Day 74 and he died on Study Day 86. Relevant medical history included prior myocardial infarction and stent placement, dyslipidemia, hypertension, and 18-year history of tobacco use. The event was considered unrelated to study treatment by the investigator.

#### Application site adverse events

Application site AEs were reported in 35 of the 190 (18.4 %) patients who experienced treatment-emergent AEs (8.1 % of total study population); no application site AEs were serious (Table 3). The highest incidence was in the vehicle QD group (Table 3). The most frequently reported application site AEs by MedDRA preferred term were psoriasis (reported by 18 [4.2 %] patients), pruritus (9 [2.1 %]), and application site pain (3 [0.7 %]).

A total of 12 (2.8 %) patients discontinued the study due to application site AEs; seven were from the vehicle QD group, three from the 2 % tofacitinib QD group, and one from each of the vehicle BID and 1 % tofacitinib QD groups. The most common application site AE leading to discontinuation was psoriasis, which was reported by six (1.4 %) patients.

#### Laboratory assessments

Thirteen patients met the criteria for laboratory safety monitoring (Table 4); no patients met the laboratory monitoring criteria for discontinuation.

**Table 4:** Patients with laboratory values meeting pre-specified protocol criteria* for safety monitoring

Criterion, % (n/N)	2 % tofacitinib BID	1 % tofacitinib BID	Vehicle BID	2 % tofacitinib QD	1 % tofacitinib QD	Vehicle QD
Any criterion	2.9 (2/70)	2.9 (2/70)	1.4 (1/69)	5.8 (4/69)	4.1 (3/73)	1.4 (1/73)
Hemoglobin^a^	1.5 (1/68)	0.0 (0/70)	0.0 (0/69)	0.0 (0/67)	4.1 (3/73)	1.4 (1/72)
Neutrophil count^b^	0.0 (0/68)	0.0 (0/69)	0.0 (0/69)	1.5 (1/67)	0.0 (0/73)	0.0 (0/72)
Lymphocyte count^c^	0.0 (0/68)	0.0 (0/70)	0.0 (0/69)	0.0 (0/67)	0.0 (0/73)	0.0 (0/71)
Platelet count^d^	0.0 (0/68)	0.0 (0/69)	0.0 (0/69)	0.0 (0/67)	0.0 (0/73)	0.0 (0/72)
Serum creatinine^e^	1.4 (1/69)	0.0 (0/70)	0.0 (0/69)	1.5 (1/67)	0.0 (0/73)	0.0 (0/72)
AST/ALT^f^	0.0 (0/69)	2.9 (2/70)	1.4 (1/69)	1.5 (1/67)	0.0 (0/73)	0.0 (0/72)
CPK^g^	0.0 (0/69)	0.0 (0/70)	1.4 (1/69)	3.0 (2/67)	0.0 (0/73)	0.0 (0/72)

^*a^Any hemoglobin value >2 g/dL (>20 g/L) below baseline; ^b^Absolute neutrophil count <1.2 × 10^9^/L (<1200/mm^3^); ^c^Absolute lymphocyte count <0.5 × 10^9^/L (<500 lymphocytes/mm^3^); ^d^Platelet count <100 × 10^9^/L (<100,000/mm^3^); ^e^Serum creatinine increase >50 % over the average of screening and baseline values OR absolute increase in serum creatinine >0.5 mg/dL (>44.2 μmol/L) over the average of screening and baseline values; ^f^Any AST and/or ALT elevation ≥3 times the ULN, regardless of the total bilirubin; ^g^Any CPK >5xULN

*ALT* alanine aminotransferase, *AST* aspartate aminotransferase, *BID* twice daily, *CPK* creatine phosphokinase, *QD* once daily, *ULN* upper limit of normal

### Pharmacokinetics

Tofacitinib concentrations were above the lower limit of quantification of 0.01 ng/mL in most plasma samples, with the largest percentage of samples in the concentration range of 0.1 to <1.0 ng/mL (Additional file 1: Table S2). There was a general trend toward higher concentrations with higher dose strength (2 % vs 1 %) but no clear difference between the dosing regimens (BID vs QD). Across tofacitinib treatment groups, 83.3 %–97.4 % of plasma tofacitinib concentrations were <1.0 ng/mL. The maximum observed plasma concentration of 9.7 ng/mL occurred at Week 12 in the 2 % tofacitinib QD group. Based on the post-dose PK obtained in a limited number of patients, the PK had a flat profile with limited fluctuation in concentrations between doses, as would be expected after topical application. Total exposure based on area under the plasma concentration time profile from time zero to the time tau (AUC_tau_) in patients with post-dose PK was higher with the higher dose strength, while the relationship between exposure and dose regimen was not clear (Additional file 1: Table S3).

## Discussion

Partial inhibition of JAK signaling by tofacitinib results in a multi-tiered intervention in the cycle of psoriasis pathogenesis, with direct impact on dysregulated keratinocytes, reduction in inflammatory infiltrate and, ultimately, normalization of the interleukin (IL)-23/Th17 axis [23]. Oral tofacitinib is effective in patients with moderate to severe plaque psoriasis [14–16], and evidence of efficacy has been seen in the patient with mild to moderate plaque psoriasis with a 2 % topical formulation of tofacitinib applied BID [17].

The current study assessed the efficacy and safety of two dose strengths (2 % and 1 %) of tofacitinib ointment applied either QD or BID in adult patients with mild to moderate plaque psoriasis. Greater efficacy response was generally observed with 2 % tofacitinib than 1 % tofacitinib, and overall no clear distinction in efficacy was seen between BID and QD dosing.

Greater efficacy of tofacitinib compared with vehicle was seen for more primary and secondary efficacy endpoints at Week 8 than Week 12. While it appeared that the PGA-C response of clear or almost clear and ≥2 grade improvement from baseline plateaued after Week 8, the vehicle treatment group PGA-C responses continued to improve after Week 8, thereby decreasing the difference between tofacitinib and vehicle at Week 12. Explanation for the increase in vehicle responses between Week 8 and Week 12 was not evident after thorough review of the study data for potential contributing factors, although it is possible this could be related to the small sample size of the study.

Patients were required to achieve a ≥2 grade improvement from baseline in PGA-C in addition to having a PGA-C score of clear or almost clear to be considered a responder for the primary efficacy endpoint. This is a much more challenging threshold than the achievement of a PGA-C score of clear or almost clear alone, when a patient need only change from mild (2) to almost clear (1) to be considered a responder. This more stringent criterion is used, as a change from the low end of the mild range to the high end of the almost clear range may not represent a clinically meaningful change.

The clinical significance of objective changes in disease severity were confirmed by the improvement in patient-reported measures. Improvements in health-related quality of life, as indicated by DLQI, reflected the changes seen in PGA-C and PASI. Greater improvements in pruritus were seen compared with vehicle in both tofacitinib BID dosing groups from Day 2 of dosing and for 2 % tofacitinib QD from Day 3. The improvements in pruritus from baseline were likely clinically meaningful (defined as a LSM decrease from baseline in ISI of 2 points based on analyses conducted with oral tofacitinib therapy for psoriasis) [19] and were seen with 1 % tofacitinib BID from Day 4 through Day 14, with 2 % tofacitinib BID from Day 5 through Day 14, and with 2 % tofacitinib QD from Day 8 through Day 14 (Fig. 3a).

Previous studies have shown oral tofacitinib improved patient-reported pruritus in moderate to severe psoriasis [19, 24, 25]. This is a direct effect, independent from improvements in clinician-reported signs of psoriasis severity [24], with a statistically significant improvement occurring as early as the second day of dosing [26]. Topical tofacitinib also improves pruritus in patients with atopic dermatitis [27].

A very rapid reduction in ISI was seen on initiation of treatment, with a significant reduction in pruritus with both 2 % and 1 % tofacitinib BID compared with vehicle BID as early as the day following the initial dose. Although pruritus is a common feature of psoriasis, the underlying pathogenesis is not understood. Impaired innervation and neuropeptide imbalance in psoriatic skin may be involved; other potential mechanisms include increased expression of IL-2, the opioid system, prostanoids, IL-31, serotonin, proteases and/or vascular abnormalities [28, 29]. Tofacitinib inhibition of JAK may suppress pruritus by blocking signaling via IL-31 [30, 31] and reducing expression of IL-2 [32]. As neuropeptides have a role in the pathogenesis of both psoriasis and pruritus, increased expression of substance P receptor, high-affinity nerve growth factor receptor or calcitonin gene-related peptide receptor may be involved [29].

Overall, topically administered tofacitinib had an acceptable safety profile, with no clinically meaningful differences in the incidence of AEs or SAEs between tofacitinib and vehicle treatment groups. The incidence of AEs coding to the MedDRA Infections and Infestations system organ class was higher in patients receiving vehicle than patients receiving tofacitinib. None of the side effects associated with topical application of potent corticosteroids were observed.

The range of observed plasma concentrations of tofacitinib from the PK analysis showed significant overlap between the dose strengths and regimens. Both AUC_tau_ and maximum observed plasma concentration were higher in patients with QD administration than with BID administration, which was not expected. In addition to dose strength and regimen, PK exposure is likely related to the treatment BSA and/or ointment application rate and this may be contributing to the lack of clear differentiation between the regimens.

In Phase 3 studies of oral tofacitinib in patients with moderate to severe psoriasis, serious infections and herpes zoster infections were associated with tofacitinib treatment [14, 15]. Based on an exposure-response analysis of oral tofacitinib psoriasis data, an average tofacitinib exposure of 12.4 ng/mL was not associated with increased incidence rates of serious infections and herpes zoster infections when compared to patients treated with placebo (unpublished observations). In the current study, more than 83 % of tofacitinib levels measured in plasma from patients in all active treatment groups were <1.0 ng/mL, which represents a >12-fold margin to the exposure levels for oral tofacitinib with no increased incidence rates for serious and herpes zoster infections relative to placebo observed in the oral tofacitinib Phase 3 psoriasis program.

### Study limitations

To form the basis for further clinical development, this Phase 2b estimation study used the 2-sided 80 % confidence interval as the pre-specified confidence level per study protocol, whereas in a Phase 3 trial the more rigorous 95 % confidence interval or 0.05 significance level would be used. The stringent eligibility criteria of a Phase 2b clinical study generally exclude some patients who may have been considered for topical treatment outside of the clinical trial environment. The numbers of patients included in the study (~70 per treatment group) is a relatively small sample size. Caution is therefore needed in extrapolating findings to real-world clinical practice. No active comparator to tofacitinib was included so efficacy was not assessed relative to another agent with a known therapeutic effect in psoriasis. No formal statistical comparison was made between QD and BID application, as study sites were assigned to either QD or BID regimens, not both. As such, the dosing regimens essentially represent two separate sub-studies.

## Conclusions

This small Phase 2b study demonstrated that topical treatment with the JAK inhibitor tofacitinib in an ointment formulation provided improvement in the clinical signs of psoriasis for patients with mild to moderate chronic plaque psoriasis. Based on the prespecified primary efficacy endpoint, which assesses clinical signs, tofacitinib as a 2 % ointment formulation applied either QD or BID showed significantly greater efficacy compared with vehicle at Week 8, but not at Week 12, and not as a 1 % ointment formulation. Acceptable safety and local tolerability profiles for both QD and BID dosing regimens were observed during 12 weeks of treatment.

## Additional files

Additional file 1:
**Figure S1.** Change from baseline in DLQI through Week 16. **Table S1.** Serious adverse events by treatment group. **Table S2.** Number and percent of plasma tofacitinib concentration samples (pre-dose and post-dose) by concentration range – available data mild/moderate - post hoc. **Table S3.** Summary of plasma tofacitinib pharmacokinetic parameters - available data at week 4 - mild/moderate - post hoc. (DOCX 238 kb) Additional file 2: List of independent ethics committees or institutional review boards. (DOCX 31 kb)

## Abbreviations

AE: Adverse event
ALT: Alanine aminotransferase
AST: Aspartate aminotransferase
AUC_tau_: Area under the plasma concentration time profile from time zero to the time tau
BID: Twice daily
BMI: Body mass index
BSA: Body surface area
CI: Confidence interval
CPK: Creatine phosphokinase
DLQI: Dermatology Life Quality Index
IL: Interleukin
ISI: Itch Severity Item
JAK: Janus kinase
LSM: Least squares mean
MedDRA: Medical Dictionary for Regulatory Activities
PASI: Psoriasis Area and Severity Index
PASI75: Psoriasis Area and Severity Index 75
PGA-C: Calculated Physician’s Global Assessment
PGA-Cm: Calculated Physician’s Global Assessment of patients with mild to moderate plaque psoriasis at baseline
PK: Pharmacokinetic
QD: Once daily
SAE: Serious adverse event
SD: Standard deviation
SE: Standard error
ULN: Upper limit of normal
