# Supplementary material for: Treatment of plaque psoriasis with an ointment formulation of the Janus kinase inhibitor, tofacitinib: a Phase 2b randomized clinical trial
Source: BMC Dermatol. 2016 Oct 3;16:15. doi: 10.1186/s12895-016-0051-4 (PMC5048458; doi:10.1186/s12895-016-0051-4)
Supplement: Additional file 1: — Figure S1. Change from baseline in DLQI through Week 16. Table S1. Serious adverse events by treatment group. Table S2. Number and percent of plasma tofacitinib concentration samples (pre-dose and post-dose) by concentration range – available data mild/moderate - post hoc. Table S3. Summary of plasma tofacitinib pharmacokinetic parameters - available data at week 4 - mild/moderate - post hoc. (DOCX 238 kb) [file 12895_2016_51_MOESM1_ESM.docx]

**Supplementary Figure 1. Change from baseline in DLQI through Week 16**

**
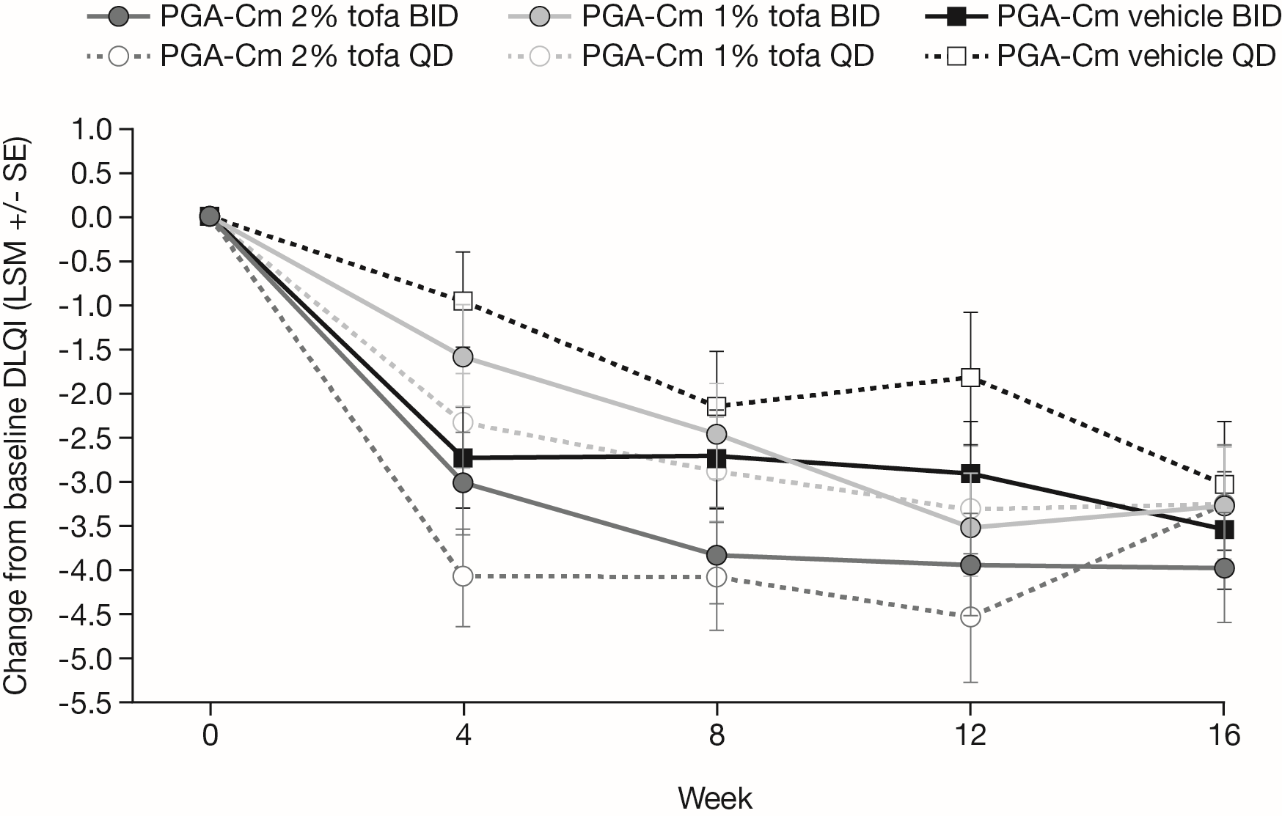
**

Least squares mean (SE) change from baseline in DLQI through Week 16 for patients applying 2% tofacitinib, 1% tofacitinib, or vehicle, once daily or twice daily. Changes from baseline were analyzed using a Mixed Model for Repeated Measures without imputation for missing values; QD and BID data were analyzed separately.

BID, twice daily; DLQI, Dermatology Life Quality Index; LSM, least squares mean; PGA-Cm, Calculated Physician’s Global Assessment of patients with mild to moderate plaque psoriasis at baseline; QD, once daily; SE, standard error; tofa, tofacitinib

**Supplementary Table 1. Serious adverse events by treatment group**

|  | **2% tofacitinib BID** | **1% tofacitinib BID** | **Vehicle**  **BID** | **2% tofacitinib QD** | **1% tofacitinib QD** | **Vehicle**  **QD** |
| --- | --- | --- | --- | --- | --- | --- |
| **MedDRA preferred term, n (%)** | **N=71** | **N=70** | **N=71** | **N=70** | **N=74** | **N=74** |
| Abdominal wound dehiscence |  | 1 (1.4) |  |  |  |  |
| Cardiac failure congestive |  | 1 (1.4) |  |  |  |  |
| Diabetes mellitus |  | 1 (1.4) |  |  |  |  |
| Myocardial infarction |  | 1 (1.4) |  |  |  |  |
| Non-cardiac chest pain |  | 1 (1.4) |  |  |  |  |
| Osteoarthritis |  |  | 1 (1.4) |  |  |  |
| Psoriatic arthropathy |  |  | 1 (1.4) |  |  |  |
| Arrhythmia |  |  |  |  | 1 (1.4) |  |
| Systemic inflammatory response syndrome |  |  |  |  | 1 (1.4) |  |
| Urinary tract infection |  |  |  |  | 1 (1.4) |  |
| Atrial fibrillation |  |  |  |  |  | 1 (1.4) |

BID, twice daily; QD, once daily; MedDRA, Medical Dictionary for Regulatory Activities

**Supplementary Table 2. Number and percent of plasma tofacitinib concentration samples (pre-dose and post-dose) by concentration range – available data mild/moderate - post hoc**

| **Tofacitinib concentration range** | **2% tofacitinib BID, n (%)** | **1% tofacitinib BID, n (%)** | **2% tofacitinib QD, n (%)** | **1% tofacitinib QD, n (%)** |
| --- | --- | --- | --- | --- |
| <0.01 ng/mL (LLOQ) | 12 (4.6) | 16 (6.0) | 6 (2.3) | 17 (5.1) |
| 0.01 to <0.1 ng/nL | 43 (16.5) | 95 (35.6) | 40 (15.2) | 77 (23.2) |
| 0.1 to <1.0 ng/mL | 164 (63.1) | 149 (55.8) | 173 (65.5) | 198 (59.6) |
| ≥ 1.0 ng/mL | 41 (15.8) | 7 (2.6) | 45 (17.0) | 40 (12.0) |
| Total | 260 (100.0) | 267 (100.0) | 264 (100.0) | 332 (100.0) |

The LLOQ was 0.0100 ng/mL

BID, twice daily; LLOQ, lower limit of quantification; n, number of all tofacitinib samples (pre-dose and post-dose) excluding Day 1 pre-dose; QD, once daily

**Supplementary Table 3. Summary of plasma tofacitinib pharmacokinetic parameters - available data at week 4 - mild/moderate - post hoc**

|  | **Parameter summary statistics^a^** | | | |
| --- | --- | --- | --- | --- |
| **Parameter (units)** | **2% tofacitinib BID** | **1% tofacitinib BID** | **2% tofacitinib QD** | **1% tofacitinib QD** |
| N | 7 | 7 | 8 | 19 |
| AUC_tau_ (ng.hr/mL) | 2.975 (187) | 1.060 (283) | 9.803 (45) | 8.944 (151) |
| C_max_ (ng/mL) | 0.3488 (222) | 0.1606 (140) | 0.5128 (54) | 0.5488 (147) |

AUC_tau_, area under the plasma concentration time profile from time zero to the time tau (τ); BID, twice daily; C_max_, maximum observed plasma concentration;

N, number of subjects contributing to the summary statistics; QD, once daily

^a^Geometric mean (geometric %CV)
