# Supplementary material for: Treatment of plaque psoriasis with an ointment formulation of the Janus kinase inhibitor, tofacitinib: a Phase 2b randomized clinical trial
Source: BMC Dermatol. 2016 Oct 3;16:15. doi: 10.1186/s12895-016-0051-4 (PMC5048458; doi:10.1186/s12895-016-0051-4)
Supplement: Additional file 2: — List of independent ethics committees or institutional review boards. (DOCX 31 kb) [file 12895_2016_51_MOESM2_ESM.docx]

**LIST OF INDEPENDENT ETHICS COMMITTEES OR INSTITUTIONAL REVIEW BOARDS**

| **Investigator** | **Ethics Committee/Institutional Review Board** |
| --- | --- |
| **Canada** |  |
| Dr. Charles W Lynde | IRB Services  suite 300  372 Hollandview Trail  Aurora, ON L4G 0A5  CANADA |
| Dr. Kim A Papp | Research Review Board  Suite 203  19 - 13085 Yonge Street  Richmond Hill, ON L4E 0K2  CANADA |
| Dr. Lyn Guenther | IRB Services  Suite 300  372 Hollandview Trail  Aurora, ON L4G OA5  CANADA |
| Dr. Marni C Wiseman | The University of Manitoba BREB  P126 - 770 Bannatyne Avenue  Pathology Building  Winnipeg, MB R3E 0W3  CANADA |
| Dr. Melinda J Gooderham | Research Review Board Inc.  Suite 203  19 - 13085 Yonge Street  Richmond Hill, ON L4E 0K2  CANADA |
| Dr. Norman R Wasel | Research Ethics Review Committee  Alberta Innovates-Health Solutions  1500, 10104-103 Avenue Northwest  Edmonton, AB T5J 4A7  CANADA |
| Dr. Rodion Kunynetz | Research Review Board Inc.  Suite 203  19 - 13085 Yonge Street  Richmond Hill, ON L4E 0K2  CANADA |
| Dr. Ronald Vender | IRB Services  Suite 300  372 Hollandview Trail  Aurora, ON L4G 0A5  CANADA |
| Dr. Wayne Carey | IRB Services  Suite 300  372 Hollandview Trail  Aurora, ON L4G 0A5  CANADA |
| Dr. Darryl P Toth | Research Review Board Inc.  Suite 203  19 - 13085 Yonge Street  Richmond Hill, ON L4E 0K2  CANADA |
| Dr. David N Adam | Research Review Board  Suite 203  19 - 13085 Yonge Street  Richmond Hill, ON L4E 0K2  CANADA |
| Dr. Chantal Bolduc | IRB Services  Suite 300  372 Hollandview Trail  Aurora, ON L4G 0A5  CANADA |
| **Denmark** |  |
| Dr. Aksel Otkjaer | Videnskabsetiske Komiteer for Region Midtjylland  Sundhedssekretariatet Skottenborg 26  Viborg, 8800  DENMARK |
| Dr. Lars Iversen | Videnskabsetiske Komiteer for Region Midtjylland  Sundhedssekretariatet Skottenborg 26  Viborg, 8800  DENMARK |
| **Poland** |  |
| Prof. Zygmunt Adamski | Komisja Bioetyczna przy Dolnoslaskiej Izbie Lekarskiej  ul. Kazimierza Wielkiego 45  Wroclaw, 50-077  POLAND |
| Dr. Jolanta Weglowska | Komisja Bioetyczna przy Dolnoslaskiej Izbie Lekarskiej  ul. Kazimierza Wielkiego 45  Wroclaw, 50-077  POLAND |
| Dr. Dorota Wielowieyska-Szybinska | Komisja Bioetyczna przy Dolnoslaskiej Izbie Lekarskiej  ul. Kazimierza Wielkiego 45  Wroclaw, 50-077  POLAND |
| Dr. Malgorzata Janczylo-Jankowska | Komisja Bioetyczna przy Dolnoslaskiej Izbie Lekarskiej  ul. Kazimierza Wielkiego 45  Wroclaw, 50-077  POLAND |
| Prof. Witold Owczarek | Komisja Bioetyczna przy Dolnoslaskiej Izbie Lekarskiej  ul. Kazimierza Wielkiego 45  Wroclaw, 50-077  POLAND |
| Dr. Marcin Ambroziak | Komisja Bioetyczna przy Dolnoslaskiej Izbie Lekarskiej  ul. Kazimierza Wielkiego 45  Wroclaw, 50-077  POLAND |
| Dr. Maria Juszkiewicz-Borowiec | Komisja Bioetyczna przy Dolnoslaskiej Izbie Lekarskiej  ul. Kazimierza Wielkiego 45  Wroclaw, 50-077  POLAND |
| Dr.Maria Czubek | Komisja Bioetyczna przy Dolnoslaskiej Izbie Lekarskiej  ul. Kazimierza Wielkiego 45  Wroclaw, 50-077  POLAND |
| Dr. Dorota Bystrzanowska | Komisja Bioetyczna przy Dolnoslaskiej Izbie Lekarskiej  ul. Kazimierza Wielkiego 45  Wroclaw, 50-077  POLAND |
| Dr. Stefan Daniluk | Komisja Bioetyczna przy Dolnoslaskiej Izbie Lekarskiej  ul. Kazimierza Wielkiego 45  Wroclaw, 50-077  POLAND |
| **United States** |  |
| Dr. Daniel M Stewart | Quorum Review IRB  1601 5th Ave, Suite 1000  Seattle, WA 98101  UNITED STATES |
| Dr. Ellen H Frankel | Quorum Review Institutional Review Board Incorporated  Suite 1000  1601 Fifth Avenue  Seattle, WA 98101  UNITED STATES |
| Dr. Alexandra B Kimball | Partners Human Research Committee  Suite 1002  116 Huntington Avenue  Boston, MA 02116  UNITED STATES |
| Dr. Alice Gottlieb | Western Institutional Review Board  3535 Seventh Avenue SW  Olympia, WA 98502  UNITED STATES |
| Dr. Angela Moore | Quorum Review Institutional Review Board Incorporated  Suite 1000  1601 Fifth Avenue  Seattle, WA 98101  UNITED STATES |
| Dr. Dowling B Stough | Quorum Review IRB  1601 5th Ave, Suite 1000  Seattle, WA 98101  UNITED STATES |
| Dr. George J Schmieder | Quorum Review IRB  Suite 1000  1601 Fifth Avenue  Seattle, WA 98101  UNITED STATES |
| Dr. Mark R Ling | Quorum Review Institutional Review Board, Inc.  Suite 800  1501 Fourth Avenue  Seattle, WA 98101  UNITED STATES |
| Dr. Howard L Sofen | Quorum Review IRB  Suite 1000  1601 Fifth Street  Seattle, WA 98101  UNITED STATES |
| Dr. Jamie Weisman | Quorum Review Institutional Review Board Incorporated  Suite 1000  1601 5th Avenue  Seattle, WA 98101  UNITED STATES |
| Dr. Jeffrey Crowley | Quorum Review IRB  1601 5th Ave, Suite 1000  Seattle, WA 98101  UNITED STATES |
| Dr. Jennie J Muglia | The Committee on the Protection of Human Subjects  593 Eddy Street  Providence, RI 02903  UNITED STATES |
| Dr. Jennifer Soung | University of California Irvine IRB  Suite 150  5171 California Avenue  Irvine, CA 92697  UNITED STATES |
| Dr. John M Humeniuk | Quorum Review Institutional Review Board Incorporated  Suite 1000  1601 Fifth Avenue  Seattle, WA 98101  UNITED STATES |
| Dr. Steven R Feldman | Wake Forest University Health Sciences IRB  Medical Center Boulevard  Winston-Salem, NC 27157  UNITED STATES |
| Dr. med. Panayiotis E  Vasiloudes | Quorum Review Institutional Review Board Incorporated  Suite 1000  1601 Fifth Avenue  Seattle, WA 98101  UNITED STATES |
| Dr. Richard L Beasley | Quorum Review IRB  Suite 1000  1601 Fifth Avenue  Seattle, WA 98101  UNITED STATES |
| Dr. Zoe D Draelos | Quorum Review Institutional Review Board Incorporated  Suite 1000  1601 Fifth Avenue  Seattle, WA 98101  UNITED STATES |
| Dr. Suzanne Bruce | Quorum Review IRB  1601 5th Ave, Suite 1000  Seattle, WA 98101  UNITED STATES |
| Dr. Tiffani K Hamilton | Quorum Review IRB  Suite 1000  1601 5th Ave  Seattle, WA 98101  UNITED STATES |
| Dr. William Abramovits | Quorum Review IRB  1601 5th Ave, Suite 1000  Seattle, WA 98101  UNITED STATES |
| Dr. Andrew Blauvelt | Quorum Review IRB  Suite 1000  1601 Fifth Avenue  Seattle, WA 98101  UNITED STATES |
| Dr. Martin A Menter | Quorum Review IRB  1601 5th Ave, Suite 1000  Seattle, WA 98101  UNITED STATES |
| Dr. Paul Getz | Quorum Review Institutional Review Board Incorporated  Suite 1000  1601 Fifth Avenue  Seattle, WA 98101  UNITED STATES |
| Dr. Mark S Lee | Quorum Review IRB  1601 5th Ave, Suite 1000  Seattle, WA 98101  UNITED STATES |
| Dr. Phoebe Rich | Quorum Review IRB  1601 5th Ave, Suite 1000  Seattle, WA 98101  UNITED STATES |
| Dr. Michael J Noss | Quorum Review Institutional Review Board Incorporated  Suite 1000  1601 Fifth Avenue  Seattle, WA 98101  UNITED STATES |
| Dr. David H Horowitz | Quorum IRB  Suite 800  1501 Fourth Avenue  Seattle, WA 98101  UNITED STATES |
